# Acute Endothelial Graft Rejection Following COVID-19 Infection

**DOI:** 10.7759/cureus.19084

**Published:** 2021-10-27

**Authors:** Geeta Behera, Tanmay Gokhale, Krishna R Babu

**Affiliations:** 1 Ophthalmology, Jawaharlal Institute of Postgraduate Medical Education and Research (JIPMER), Puducherry, IND

**Keywords:** sars-cov-2, covid-19, acute endothelial graft rejection, therapeutic penetrating keratoplasty, corneal ulcer

## Abstract

Here, we report a case of acute endothelial graft rejection following coronavirus disease 2019 (COVID-19). A 57-year-old woman who underwent therapeutic penetrating keratoplasty for a perforated infectious corneal ulcer in her right eye developed severe acute respiratory syndrome coronavirus 2 infection, which required intensive care and treatment with steroids. Acute endothelial graft rejection was seen at three weeks postoperatively and managed with high-dose corticosteroids. Despite standard therapy, secondary graft failure was observed. Immune dysregulation associated with COVID-19 may be a significant cause of acute endothelial graft rejection among keratoplasty patients with COVID-19.

## Introduction

The coronavirus disease 2019 (COVID-19) caused by severe acute respiratory syndrome coronavirus 2 (SARS-CoV-2) was initially thought to affect the respiratory tract and the lung parenchyma. However, it has now been demonstrated to be a multisystem disease with complex interactions with coexisting medical conditions and causing indirect effects through immune dysregulation [1]. An unusually high rejection rate among pancreatic and renal transplant recipients in the setting of COVID-19 suggests sensitization to allogenic graft antigens mediated by widespread immune reactivation despite immunosuppressants [2]. However, immune response activation in the eye following COVID-19 infection in corneal transplant patients is not expected to be as common, presumably because the cornea is an immune-privileged site [3]. There are very few reports of corneal graft rejection post-COVID-19 infection to date [4,5]. Here, we report a case of acute corneal graft rejection post-COVID-19 infection.

## Case presentation

**Figure 1:**
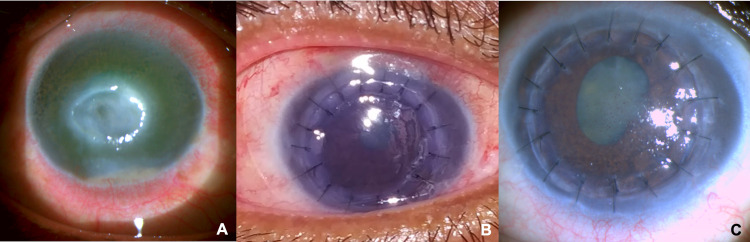
Slit-lamp images. (A) Central corneal ulcer measuring 4.0 × 5.5 mm with stromal thinning and a hypopyon measuring 1.8 mm. (B) Clear corneal graft at three weeks postoperatively. (C) Severe corneal graft edema, with pigments/granulomatous keratic precipitates and posterior synechiae at the fourth week.

A 57-year-old woman, an agricultural laborer, presented with acute-onset painful diminution of vision in the right eye for 10 days following injury with vegetative matter while working. The best-corrected visual acuity (BCVA) at presentation was light perception (PL). On examination, she had a central corneal ulcer measuring 4.0 × 5.5 mm with stromal thinning and a hypopyon measuring 1.8 mm (Figure 1, Panel A). There was no view of the fundus, but B-scan ultrasonography of the eye was normal. Intraocular pressure (IOP) could not be measured. On examination, the left eye was normal (BCVA: 6/6, IOP: 14 mmHg). Initial corneal scrapings for microscopy showed pus cells, but no organism was isolated. We started medical management with hourly topical gatifloxacin (0.5%), tobramycin (1.3%), and topical natamycin (5%). However, the ulcer perforated on the fourth day. It was a central perforation in a small area of extreme thinning. Hence, penetrating keratoplasty with a wide margin (1 mm) from the infected tissue was performed: host cut, 7.0 mm; graft cut, 7.5 mm (donor endothelial count: 3,076 cells/mm^2^). The graft was clear postoperatively with an epithelial erosion of 7 × 5 mm, with a well-formed anterior chamber [BCVA: counting fingers close to face, IOP: 12 mmHg]. Post-keratoplasty, she received topical natamycin (5%), topical moxifloxacin (0.5%) and dexamethasone (0.1%) combination four times a day, topical homatropine (2%) twice a day, topical lubricants (carboxymethylcellulose 0.5%) hourly, and oral prednisolone (1 mg/kg/day).

On the second postoperative day, the patient complained of nausea, vomiting, cough, and mild breathlessness. She tested positive for COVID-19 by reverse transcription-polymerase chain reaction and was isolated. Her oxygen saturation was 92%. An X-ray of the chest on posteroanterior view showed ground-glass opacities, suggestive of COVID-19-associated pneumonia. She was isolated and treated with intravenous dexamethasone (6 mg/day), subcutaneous enoxaparin (60 mg/day), and supplemental oxygen via a mask (4 L/minute) for five days by the Internal Medicine department. However, we continued her postoperative care. An ophthalmology resident reviewed her daily with appropriate personal protection and ensured compliance with postoperative treatment. A microbiological analysis reported *Candida* spp. sensitive to voriconazole and amphotericin B from the excised corneal button. Topical natamycin was stopped (day 10), and topical voriconazole (1%) was started with the continuation of topical moxifloxacin (0.5%) and dexamethasone (0.1%) combination four times a day, and topical lubricants (carboxymethylcellulose 0.5%) hourly. The epithelial defect healed completed by the eighth day. The graft cornea was clear for three weeks postoperatively with no evidence of reactivation of the fungal keratitis or graft failure. (Figure 1, Panel B)

However, the patient had severe corneal graft edema at subsequent review, with pigments and granulomatous keratic precipitates (KPs), posterior synechiae, inflammatory iris nodules 3+, and anterior chamber cells, suggestive of acute graft rejection (BCVA: PL, IOP: 18 mmHg) (Figure 1, Panel C). Hence, we administered a single dose of intravenous methylprednisolone (1 g) followed by oral prednisolone (1 mg/kg/day, tapered by 10 mg/week), topical moxifloxacin (0.5%) and dexamethasone (0.1%) combination hourly. and topical lubricants (carboxymethylcellulose 0.5%) hourly. The anterior chamber reaction, pigments, and KPs were reduced by the fifth day, and a tapered regimen of topical and systemic steroids was administered. The patient did not develop exudates, infiltrates, or hypopyon, indicative of persistent infection at any point in time. At three months postoperatively, graft edema (secondary graft failure) and pigmented KPs persisted [BCVA: hand movement, IOP: 20 mmHg]. She is currently on the corneal transplant waiting list.

## Discussion

Immune dysregulation in primary infection by SARS-CoV-2 has the potential to incite fatal immune responses. Immune reactivation to allogeneic antigens has also been noted among renal and pancreatic transplant recipients following COVID-19 [6]. The general pro-inflammatory state caused by the disease contributes to the breach of the immune privilege of the cornea. Two previously reported cases of acute endothelial rejection coinciding with or following COVID-19 support the hypothesis of immune dysregulation overwhelming the blood-aqueous barrier, inciting an acute rejection reaction [4,5]. Though these patients were at low risk of rejection, one of them was on a very low-dose topical steroid (once daily maintenance dose), which may have been inadequate to prevent the event [5]. The maintenance therapy-related information is not available for the second case [4].

Corneal graft rejection has also been reported following BNT162b2 mRNA SARS-CoV-2 (BioNTech/Pfizer) and ChAdOx1 nCoV-19 (Covishield, Oxford AstraZeneca, United Kingdom, manufactured by the Serum Institute of India) vaccination [6-8]. In all but one of the reported five cases, the topical steroid was either discontinued or reduced to just once daily maintenance dosage as the patients had a stable course [6-8]. Only one patient developed acute graft rejection 21 days after Descemet’s membrane endothelial keratoplasty seven days following BNT162b2 mRNA SARS-CoV-2 (BioNTech/Pfizer) vaccination, with the timing of rejection similar to ours [6]. The authors postulated that the direct pathway triggered the rejection in their case, which may also be the mechanism in our case [6].

Our patient underwent therapeutic keratoplasty for infectious keratitis, in which higher rejection rates have been reported (27.3%) [9]. The rejection is related to acute inflammation caused by the infection and the use of large or eccentric grafts. The latter does not apply to our case because the patient had a central perforation and we performed a standard-sized keratoplasty. Additionally, our patient received high doses of systemic steroids as part of the management protocol for COVID-19 pneumonia and was subsequently continued on a slow taper of oral steroids. It may be noted that despite this, there was no persistent infection in our patient. We attribute it to the 1 mm wide excision of the cornea around the infected tissue. The graft rejection suggests an overwhelming hypersensitivity response that could not be suppressed by the combination of topical and systemic immunosuppressive therapy. The presence of memory T-cells that could mediate a hypersensitivity response has been demonstrated in the serum of recovered COVID-19 patients [10]. It has been used as a marker in the form of a skin test [10]. Whether the same mechanism applies to an immune-privileged site such as the eye needs to be investigated further.

Coincident COVID-19 infection might have contributed to early graft rejection, in addition to the inflammation related to infectious keratitis in our patient who underwent therapeutic keratoplasty. Based on our experience and current evidence, we recommend close monitoring of keratoplasty patients who develop COVID-19 for early recognition of rejection and salvage of the graft. Furthermore, we recommend increasing the maintenance dose of topical steroid therapy in post-keratoplasty recipients who develop COVID-19 to avoid a rejection reaction.

## Conclusions

Acute endothelial graft rejection may be triggered by COVID-19. Hence, all keratoplasty recipients who develop COVID-19 should be closely monitored, and maintenance topical steroid therapy can be increased to avoid a rejection reaction.
